# Avoiding the Banality of Evil in Times of COVID-19: Thinking Differently with a Biopsychosocial Perspective for Future Health and Social Policies Development

**DOI:** 10.1007/s42399-020-00486-8

**Published:** 2020-09-01

**Authors:** Matilde Leonardi, Haejung Lee, Sabina van der Veen, Thomas Maribo, Marie Cuenot, Liane Simon, Jaana Paltamaa, Soraya Maart, Carole Tucker, Yanina Besstrashnova, Alexander Shosmin, Daniel Cid, Ann-Helene Almborg, Heidi Anttila, Shin Yamada, Lucilla Frattura, Carlo Zavaroni, Qiu Zhuoying, Andrea Martinuzzi, Michela Martinuzzi, Francesca Giulia Magnani, Stefanus Snyman, Ahmed Amine El Oumri, Ndegeya Sylvain, Natasha Layton, Catherine Sykes, Patricia Welch Saleeby, Andrea Sylvia Winkler, Olaf Kraus de Camargo

**Affiliations:** 1 UOC Neurology, Public Health, Disability, Fondazione IRCCS Istituto Neurologico Carlo Besta, Via Celoria 11, 20133 Milan, Italy; 2 Università Cattolica del Sacro Cuore, Milan, Italy; 3 Silla University, Busan, South Korea; 4 Department of Ethics, Law and Humanities, Amsterdam UMC, VU University Amsterdam, Amsterdam, The Netherlands; 5 Department of Public Health, Aarhus University, Aarhus, Denmark; 6 DEFACTUM, Central Denmark Region, Aarhus, Denmark; 7 School of public Health, École des hautes études en santé publique (EHESP), Rennes, France; 8 MSH Medical School Hamburg, Hamburg, Germany; 9 JAMK University of Applied Sciences, Jyväskylä, Finland; 10 Department of Health and Rehabilitation Sciences, Faculty of Health Sciences, University of Cape Town, Cape Town, South Africa; 11 College of Public Health, Temple University, Philadelphia, PA USA; 12 Albrecht Federal Scientific Centre of Rehabilitation of the Disabled, St. Petersburg, Russia; 13 Centre for Innovations and Development in Healthcare (CIDEAS), Santiago del Chile, Chile; 14 National Board of Health and Welfare, Stockholm, Sweden; 15 Finnish Institute for Health and Welfare (THL), Helsinki, Finland; 16 Department of Rehabilitation Medicine, Kyorin University School of Medicine, Tokyo, Japan; 17 Strategical Directorate, Classification Area, Azienda Sanitaria Universitaria Giuliano Isontina, Trieste, Italy; 18 Research Institute of Rehabilitation Information, China Rehabilitation Research Center/WHO-FIC CC China, Beijing, China; 19 Department of Conegliano-Pieve di Soligo, IRCCS E. Medea Scientific Institute, Conegliano, Italy; 20 King’s College London, GKT School of Medical Education, London, UK; 21 Centre for Community Technologies, Nelson Mandela University, Port Elizabeth, South Africa; 22 Mohammed VI University Hospital of Oujda, Faculty of Medicine of Oujda, Mohammed First University of Oujda, Oujda, Morocco; 23 Stand Together for Change (STC), Kigali, Rwanda; 24 Rehabilitation, Ageing and Independent Living Research Centre, Monash University, Frankston, Victoria Australia; 25 University of Sydney, Sydney, Australia; 26 Department of Sociology, Criminology, and Social Work, Bradley University, Peoria, IL USA; 27 Center for Global Health, Department of Neurology, Technical University of Munich, Munich, Germany; 28 Centre for Global Health, Institute of Health and Society, University of Oslo, Oslo, Norway; 29 CanChild - Centre for Childhood Disability Research, McMaster University, Hamilton, Ontario Canada

**Keywords:** COVID-19, SARS-CoV-2, Disability, Public health, Health policies

## Abstract

The COVID-19 pandemic provides the opportunity to re-think health policies and health systems approaches by the adoption of a biopsychosocial perspective, thus acting on environmental factors so as to increase facilitators and diminish barriers. Specifically, vulnerable people should not face discrimination because of their vulnerability in the allocation of care or life-sustaining treatments. Adoption of biopsychosocial model helps to identify key elements where to act to diminish effects of the pandemics. The pandemic showed us that barriers in health care organization affect mostly those that are vulnerable and can suffer discrimination not because of severity of diseases but just because of their vulnerability, be this age or disability and this can be avoided by biopsychosocial planning in health and social policies. It is possible to avoid the banality of evil, intended as lack of thinking on what we do when we do, by using the emergence of the emergency of COVID-19 as a Trojan horse to achieve some of the sustainable development goals such as universal health coverage and equity in access, thus acting on environmental factors is the key for global health improvement.

“The term *emergence* indicates both the manifestation of something already existing, but not yet put in the foreground, but also the appearing of the unexpected, the uncalculated, the unknown, and the new” as the Italian philosopher Adriano Pessina writes “The SARS-CoV-2 (COVID-19) pandemic of today” he continues “is an emergency that fulfils both meanings: for a long time, scientists predicted an epidemic, but this coronavirus is dramatically new and therefore difficult to contrast with past experiences. Each *emergency* establishes a reordering of values, choices, and decisions. Facing the emergency means putting aside a series of problems, unsolved issues, and looking ahead. Emergencies often unleash unexpected capacities, free ethical reserves, and give rise to new ‘emergencies’” [1]. Health is a global political matter and a public good indispensable for the exercise of human rights. Health care and public health, as well as health promotion and diseases prevention, have imperatives that may conflict with economic priorities and countries’ reality. What could emerge from the COVID-19 pandemic’s lesson could also be a global common approach to health.

COVID-19 is currently threatening almost all national health systems. Each government deploys resources, trying to meet the United Nations’ Sustainable Development Goal No. 3 (Ensure healthy lives and promote well-being for all at all ages) while balancing health care expenditures within national economies. The limited resources in health care and services, which in recent years have been progressively eroded, make responding to health crises precarious. Therefore, the real challenge is how to develop a new future, able to live with new possible pandemics, embracing “the awareness of the individual and collective human rights to equally access services and ensure that all people are treated with dignity and respect” [2].

The reorganization of health and social systems after the pandemic requires the capacity to deal with the complexity of the real world. The COVID-19 pandemic disclosed an epidemic of stigma, discrimination, and prejudice against the most vulnerable: people with disabilities and chronic conditions, children, and ageing people [3–5]. The pandemic is likely to disproportionately affect these millions of individuals, putting them at greater risk of morbidity and mortality and increasing world’s disability. These experiences underscore the urgent need to improve health care focused on persons and their well-being, and maintain the global health commitment to achieving Universal Health Coverage [6] through collaboration across all government’s sectors. The guiding framework for this is the UN-Convention for the Rights of People with Disabilities [7], ratified by many countries which condemns discrimination on the base of disability or age.

To avoid that the current situation develops into a worldwide catastrophe, stable long-term solutions are required aligned with ethical principles and hard-won rights for everyone. Even in acute surge situations, triage protocols have been unevenly applied in many countries, due to the ongoing biases of the professionals who were expected to implement them [8]. The more sophisticated the triage process becomes, more precious time of health professionals will be needed in this decision-making process instead of providing care on the front lines. Therefore, in High but also in Low- and Middle-Income Countries, it must be ensured that vulnerable people do not face discrimination in the allocation of life-sustaining treatments, in the acute and long-term care [9].

Planning for the future on how to manage the complexity of resource allocation during COVID-19 times requires the capacity to think explicitly by using the biopsychosocial approach that is also the base of the WHO’s International Classification of Functioning, Disability and Health (ICF) [10]. ICF defines health by the evaluation of biological factors but also of the context in which people live and governments act. Thus, actions to improve global health could be defined at both biological and systems levels. Policies, systems, services, and attitudes must be facilitators and not barriers for people. The pandemic can serve as the Trojan horse to improve health services and to plan a new biopsychosocial based era, free of discrimination due to disability, age, or health condition.

The “banality of evil” refers to lack of thinking, as stated by the philosopher Hannah Arendt. The COVID-19 pandemic provides the opportunity to think and adopt a biopsychosocial perspective in acting on environmental factors by investing in prevention and promotion, in health personnel, in tackling avoidable health inequalities, boosting health literacy, and ensuring that social and health protection systems are solid and well-funded. Investing in these services means investing in people, resilience, solidarity, and, ultimately, in the wellbeing of the whole society. The opportunity for think and change so as to really “Leave no one behind” is now.
